# Strain-assisted magnetization reversal in Co/Ni multilayers with perpendicular magnetic anisotropy

**DOI:** 10.1038/srep27774

**Published:** 2016-06-14

**Authors:** D. B. Gopman, C. L. Dennis, P. J. Chen, Y. L. Iunin, P. Finkel, M. Staruch, R. D. Shull

**Affiliations:** 1 Materials Science and Engineering Division, NIST, Gaithersburg, MD, 20899, USA; 2 Institute of Solid State Physics, RAS, Chernogolovka, 142432, Russia; 3 U.S. Naval Research Laboratory, Washington, DC, 20375, USA

## Abstract

Multifunctional materials composed of ultrathin magnetic films with perpendicular magnetic anisotropy combined with ferroelectric substrates represent a new approach toward low power, fast, high density spintronics. Here we demonstrate Co/Ni multilayered films with tunable saturation magnetization and perpendicular anisotropy grown directly on ferroelectric PZT [Pb(Zr_0.52_Ti_0.48_)O_3_] substrate plates. Electric fields up to ±2 MV/m expand the PZT by 0.1% and generate at least 0.02% in-plane compression in the Co/Ni multilayered film. Modifying the strain with a voltage can reduce the coercive field by over 30%. We also demonstrate that alternating in-plane tensile and compressive strains (less than 0.01%) can be used to propagate magnetic domain walls. This ability to manipulate high anisotropy magnetic thin films could prove useful for lowering the switching energy for magnetic elements in future voltage-controlled spintronic devices.

Perpendicular magnetic anisotropy (PMA) continues to generate significant technological interest, particularly due to the superior thermal stability over in-plane anisotropies when scaling down materials for high density magnetic data storage and spintronics applications^1^,^2^. The non-volatility required in magnetic nano-objects for high-density storage and logic requires high anisotropy thin film materials such that the ratio of anisotropy energy to thermal energy (*KV*/*k*_*B*_*T*) is greater than 50, where *K* is the anisotropy energy density, *V* is the volume of a single magnetic nano-object, *k*_*B*_ is the Boltzmann constant and *T* is the temperature^3^,^4^. At the same time, the energy consumed by switching the magnetization must be minimized in order to realize energy efficient magnetic random access memory (MRAM) devices. One way to reduce the energy required for reversing the magnetization while maintaining sufficient magnetic anisotropy energy for thermal stability is to independently control the magnetization by a variable other than an applied external magnetic field^1^.

Magnetoelectric coupling is a promising method for this independent control of magnetization. A voltage applied to a ferroelectric substrate generates strains that can mechanically couple to a ferromagnetic element and modify its magnetic anisotropy^5^. Previous studies have explored strain-induced changes in magnetic anisotropy energy and domain wall propagation in hybrid piezoelectric/ferromagnetic heterostructures, for both in-plane and perpendicularly magnetized ferromagnetic thin films either by direct deposition^6^,^7^,^8^,^9^,^10^ of the magnetic film onto the piezoelectric element or by indirect bonding^11^,^12^ of the piezoelectric element onto a magnetic film on substrate. Among the materials showing PMA, Co/Pd, Co/Pt and CoFeB have been explored previously, largely due to their promise for high density magnetic recording and MRAM.

Co/Ni multilayers have attracted interest for their use in spin-valve and domain wall devices^13^,^14^,^15^,^16^. Until now this system has not been explored as part of such a ferromagnetic/ferroelectric heterostructure. Composed entirely of transition-metal ferromagnets, Co/Ni multilayers exhibit a tunable saturation magnetization by alternating the Co to Ni ratio and exhibit high spin-polarization due to the relatively lower spin-orbit coupling in this system compared with other PMA systems (Co/Pt, Co/Pd, FePt)^17^. Moreover, the magnetoelastic contributions to the total magnetic energy from uniaxial out-of-plane (biaxial in-plane) strain in bulk cubic (111) Co and Ni have opposite signs, which makes this a particularly interesting material system to study strain-induced changes in magnetism in ultrathin layers of cobalt and nickel^18^,^19^,^20^.

Here, we demonstrate that Co/Ni multilayers can be grown directly on top of ferroelectric Pb(Zr_0.52_Ti_0.48_)O_3_ (PZT) substrate plates. This is distinct from previous studies that bonded PZT transducers to ferromagnetic films grown on non-piezoelectric substrates, and allows direct strain coupling between overlaid film and substrate^11^,^12^. We tune the magnetization and the PMA energy by changing the relative layer thicknesses of Co and Ni and by varying the number of layer repeats. Using the Co/Ni multilayer showing the strongest PMA energy, we investigate strain-assisted magnetization reversal induced by voltages applied to the PZT substrate. We find that the voltages applied to the PZT generate strain that can couple directly to the Co/Ni multilayers and that reductions in the coercivity of the magnetic film track the PZT strain-voltage curve with a strain dependence of −1 mT per 0.15% tensile strain in the PZT, permitting one to tune the coercivity by 30%, commensurate with achieved strain-mediated coercivity reductions in other candidate materials for voltage-assisted magnetization reversal with PMA. This large modification of the magnetic coercivity caused by interfacial strain coupling shows promise for voltage-assisted magnetization reversal in Co/Ni multilayers /PZT heterostructures.

## Results

### Perpendicular Magnetic Anisotropy of Co/Ni Multilayers on PZT

The multilayered film structures used in this study are prepared on PZT substrates by dc magnetron sputtering at room temperature with a base pressure below 6.6 × 10^−6^ Pa (5 × 10^−8^ Torr). The PZT substrate is polycrystalline and 1 mm thick (see Supplementary Information), even after chemical-mechanical polishing (as confirmed with a micrometer). Co/Ni multilayers are grown on top of a seed layer of Ta(3 nm)/Pt(2 nm) and are capped by a Pt(1.6 nm)/Ta(3 nm) bilayer. For magnetization (*M*) versus applied field (*μ*_*0*_*H*) and ferromagnetic resonance (FMR) measurements, five different multilayers are prepared: [Co(0.15)/Ni(0.3)]_x16_; [Co(0.2)/Ni(0.4)]_x16_; [Co(0.2)/Ni(0.6)]_x16_; [Co(0.15)/Ni(0.6)]_x16_ and [Co(0.15)/Ni(0.6)]_x4_ (numbers in parentheses are nominal thicknesses in nm and subscripts indicate the number of bilayer repeats). For symmetry, each Co/Ni multilayered film is additionally topped with an extra cobalt layer of the same thickness as in the multilayer (e.g [Co(0.15)/Ni(0.6)]_x4_ has an added 0.15 nm cobalt layer) before the Pt/Ta capping bilayer. From x-ray diffraction, the Co/Ni multilayer was determined to be polycrystalline with an fcc crystal structure (see Supplementary Information).

**Figure 1:**
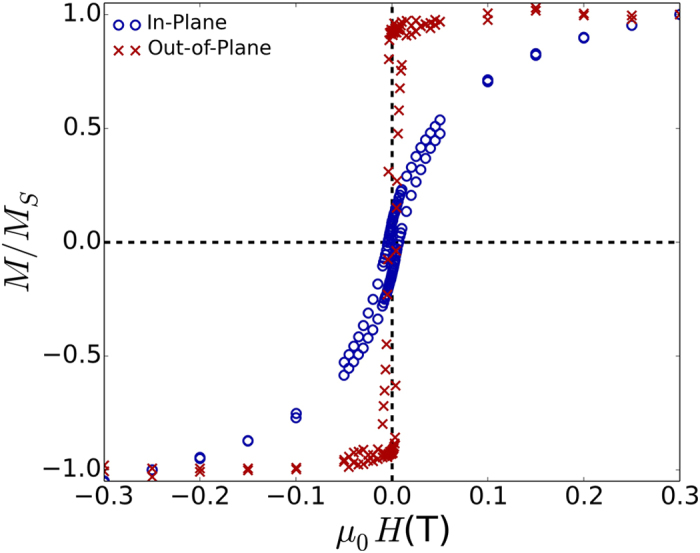
Normalized magnetization (*M*/*M*_S_) versus applied magnetic field for the [Co(0.15)/Ni(0.6)]_x16_ film showing out-of-plane easy axis. Magnetic fields are applied either in plane (open blue circles) or out-of-plane (red crosses).

**Figure 2:**
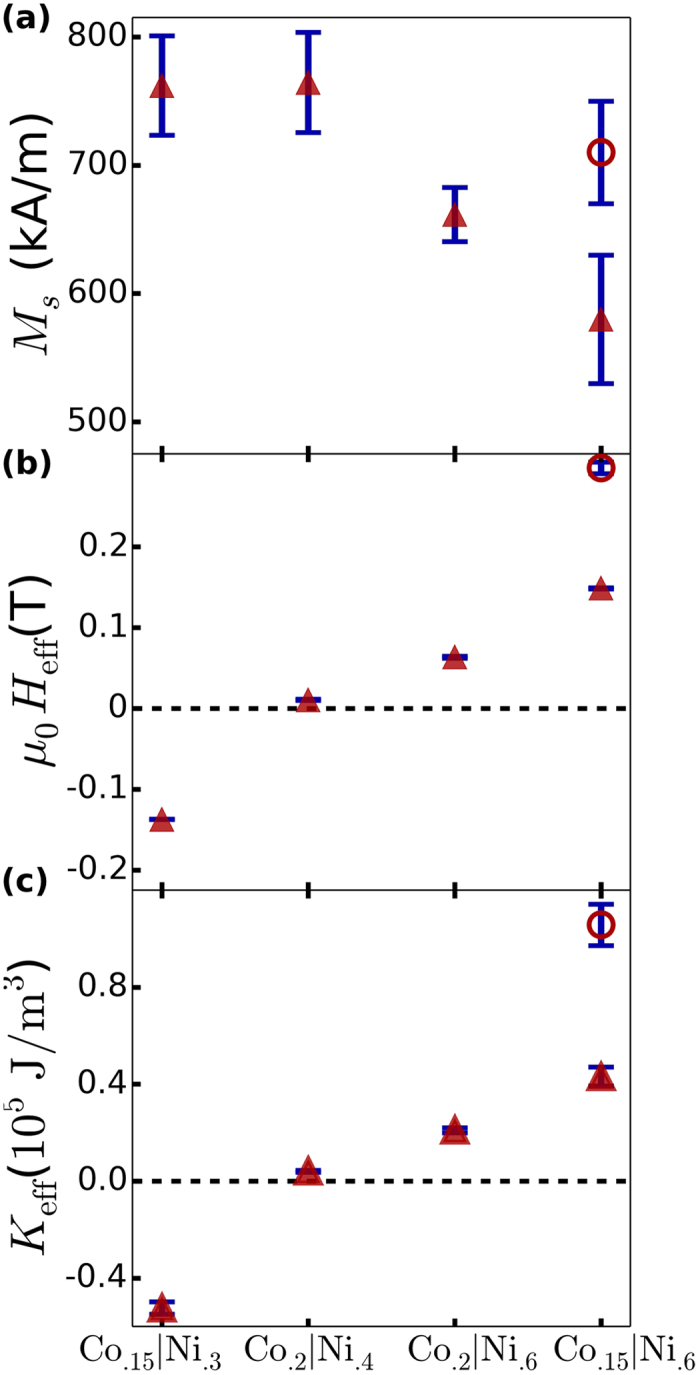
(**a**) Saturation magnetization determined from magnetization versus applied out-of-plane field hysteresis loops; (**b**) effective anisotropy field determined from ferromagnetic resonance frequency versus applied out-of-plane magnetic field and (**c**) anisotropy energy evaluated from (**a,b**) results. All solid markers represent samples containing 16 bilayer repeats of Co_x_|Ni_y_ (x,y are layer thicknesses in nm), with the unfilled round marker representing the sample consisting of 4 bilayers of Co_0.15_|Ni_0.6_.

Figure 1 shows the in-plane and out-of-plane magnetization versus external magnetic field (*M-H*) curves for the [Co(0.15)/Ni(0.6)]_x16_ film, as determined from vibrating sample magnetometry. It can be seen that the sample shows an out-of-plane easy axis when comparing the relatively higher saturation field and lower remnanent magnetization of the in-plane magnetization loop compared to the out-of-plane magnetization loop. The saturation magnetization can be tuned by adjusting the relative thicknesses of Co and Ni, as well as the number of repeats, as can be seen in Fig. 2(a). We obtain the largest saturation magnetization for the [Co(0.2)/Ni(0.4)]_x16_ film (*M*_S_ = 780 ± 40 kA/m). With increasing nickel content relative to cobalt in the bilayer period, a decrease in the saturation magnetization is observed, a trend in agreement with the lower saturation magnetization of Ni relative to Co.

The perpendicular anisotropy field *μ*_0_*H*_eff_ was estimated from fits to the out-of-plane ferromagnetic resonance field versus frequency measured between 10 GHz and 25 GHz. The out-of-plane resonance field is linearly dependent on frequency, according to the Kittel equation^21^:


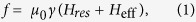



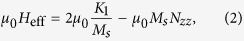


for which *f* is the microwave excitation frequency, *μ*_0_*H*_*res*_ is the resonant field, and *γ* is the gyromagnetic ratio. The effective magnetic anisotropy field (*μ*_0_*H*_eff_) comprises the surface and interface perpendicular anisotropy (*K*_1_) as well as the demagnetization field (*μ*_0_*M*_*s*_*N*_*zz*_), where the demagnetizing factor (*N*_*zz*_) for a continuous thin film is equal to one. For *μ*_0_*H*_eff_ > 0, the magnetization is oriented perpendicular to the film plane. Figure 2(b) presents *μ*_0_*H*_eff_ for the five multilayers, showing a net PMA for all samples except for the [Co(0.15)/Ni(0.3)]_x16_ film (where *μ*_0_*H*_eff_ = −137 ± 1 mT). Decreasing the Co to Ni ratio reduces the demagnetization field (*μ*_0_*M*_*s*_*N*_*zz*_) and can lead to gains in the PMA, particularly for the two Co(0.15)/Ni(0.6) multilayers. By reducing the number of Co/Ni bilayer repeats from sixteen to four, and correspondingly the overall magnetic thickness, the largest PMA (*μ*_0_*H*_eff_ = 268 ± 7 mT) is seen in the [Co(0.15)/Ni(0.6)]_x4_ film. This is likely a reflection of the relatively larger contribution (compared to total anisotropy) of the Pt/Co interfacial surface anisotropy generated in the seed and capping layers, which is known to be larger than the surface anisotropy at the Co/Ni interfaces^13^,^22^,^23^,^24^. Finally, the PMA energy *K*_eff_ = (*μ*_0_*H*_eff_*M*_s_/2) of multilayers was estimated from the anisotropy field *μ*_0_*H*_eff_ and the saturation magnetization *M*_s_ and is displayed in Fig. 2(c). For the highest PMA film, the PMA energy density *K*_eff_ = (0.95 ± 0.08) × 10^5^ J/m^3^, a value high enough for non-volatile storage (*K*_*eff*_*V*/*k*_*B*_*T* > 50) in a 3 nm thick magnetic nano-element at reduced dimensions (30 nm diameter).

### Strain-assisted magnetization reversal

**Figure 3:**
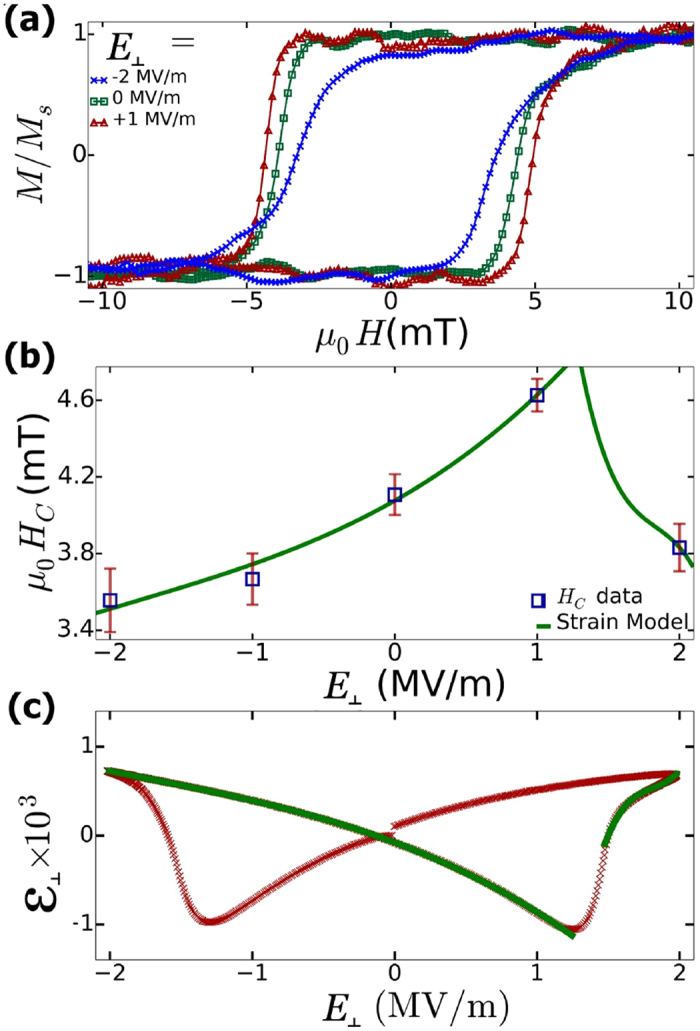
(**a**) Kerr microscopy magnetic hysteresis loops under applied electric fields to the 1 mm thick PZT substrate; **(b)** magnetic coercivity (blue open squares) versus applied electric field parameterized (green curve) by non-linear strain-electric field curve obtained for the PZT substrate in (c).

The next step is to determine the effect of strain on magnetization reversal in the [Co(0.15)/Ni(0.6)]_x4_ multilayered film which had the largest PMA. Electrical contact was made to both the top and bottom of the 1 mm thick PZT substrate in order to apply voltages and strain the PZT. We used a silver epoxy to make a conductive bond between a copper plate and the bottom of the PZT substrate. Similarly, a 0.2 mm copper wire was bonded to the top of the multilayered film to apply voltages to the top surface of the PZT. Poling of the PZT was achieved by applying +2 kV between the top and bottom surfaces of the PZT for one hour while immersed in a dielectric bath, heated to 350 K. We measured the effects of the applied voltages by characterizing the magnetic hysteresis loops using Kerr microscopy. Figure 3(a) shows out-of-plane hysteresis loops of the Co/Ni multilayers while applying electric fields of +1 MV/m, 0 and −2 MV/m through the thickness of a ceramic PZT substrate and in the out-of-plane direction (*E*_⊥_). The coercive field depends on the out-of-plane applied electric field magnitude and polarity – under a positive electric field (1 MV/m), the coercive field is maximal and then reaches a minimum for a negative electric field (−2 MV/m). In Fig. 3(b) we present a non-linear magnetic coercivity trend under a series of applied electric fields beginning at −2 MV/m and then increasing in increments of +1 MV/m increments. We observe a (repeatable) trend in which the coercivity increases with increasing positive electric fields to a point, from *μ*_0_*H*_*c*_ = 3.7 ± 0.1 mT for *E*_⊥_ = −2 MV/m up to *μ*_0_*H*_*c*_ = 4.6 ± 0.1 mT for *E*_⊥_ = +1 MV/m, followed by a decrease with further increases in the electric field to *μ*_0_*H*_*c*_ = 4.0 ± 0.1 mT for *E*_⊥_ = +2 MV/m. This (repeatable) trend reverses itself as the electric field is then swept from large positive to large negative values, with a maximum in the magnetic coercive field at *E*_⊥_ = −1 MV/m.

The non-linear relationship between the magnetic coercivity and the applied electric field is a direct consequence of the non-linear strain-voltage relationship in the PZT substrate. In Fig. 3(c), we show the strain in PZT as a function of applied electric field. Strains in the PZT along the poling direction were determined by measured displacements in a homemade linear variable differential transformer. The curve for a full sweep of the electric field is a non-linear, butterfly-shaped loop, with a maximum tensile strain in the PZT in excess of 0.15%. To better estimate the electric field-strain relationship for arbitrary electric fields, we interpolated the PZT strain-voltage butterfly curve with third order polynomials to two sections of the strain curve (e.g. from −2 MV/m to +1 MV/m and from +1.5 MV/m to +2 MV/m). Using this empirically determined parameterization on the measured magnetic coercivity versus electric field data, we find that the coercivity-voltage relationship for the Co/Ni multilayers (solid curves in Fig. 3(b)) is linearly proportional to the PZT strain-voltage relationship, which is consistent with previous results for strain-induced modification of the PMA^9^. From the strain-coercivity relationship, we extrapolate that a 0.15% tensile strain of the PZT thickness would reduce the coercivity by 1 mT.

To better understand the relationship between strain in the PZT and reduction in the Co/Ni multilayer coercivity, we begin by estimating the magnetoelastic anisotropy change associated with a 0.15% elongation of the PZT substrate thickness (corresponding to a −1.0 +/− 0.2 mT coercivity change). The bulk magnetoelastic anisotropy change for a face-centered-cubic structure, where the out-of-plane magnetization aligns with the (111) crystallographic direction, the change in magnetoelastic energy is estimated as


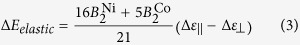


where 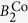 = −29 MJ/m^3^ and 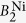 = +10 MJ/m^3^ reflect the (111) cubic magneto-elastic coupling coefficients of Co and Ni^25^. If we then assume perfect transmission of in-plane strain from the PZT (*ν*_*PZT*_ = 0.3), we arrive at 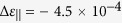, and subsequently the out-of-plane strain through elastic deformation of the Co/Ni 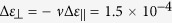, where we attribute a Poisson ratio of *ν* = 0.33 for the metallic Co/Ni multilayers^9^,^26^. From these values, we estimate a change in magnetoelastic energy of −1.3 kJ/m^3^, or a reduction in the PMA field by 1.2 mT. Although this calculated value is 20% larger than our measured coercivity change, it lends additional support to the hypothesis that the coercivity is strain-mediated, but with room for further improvements, particularly in strategies to optimize the strain coupling at the interface, such as reducing the moderate interfacial roughness between the PZT and the Co/Ni multilayers (see Supplementary Information). Furthermore, the calculated change in the PMA energy is less than a 1% reduction in the total PMA energy. This modest reduction in the PMA is expected based on the very large interfacial PMA relative to volume magnetoelastic anisotropy as has been reported previously^20^,^27^. In the following section, we will also demonstrate a meaningful contribution to the strain-mediated coercivity reduction from strain-mediated magnetic domain wall propagation.

**Figure 4:**
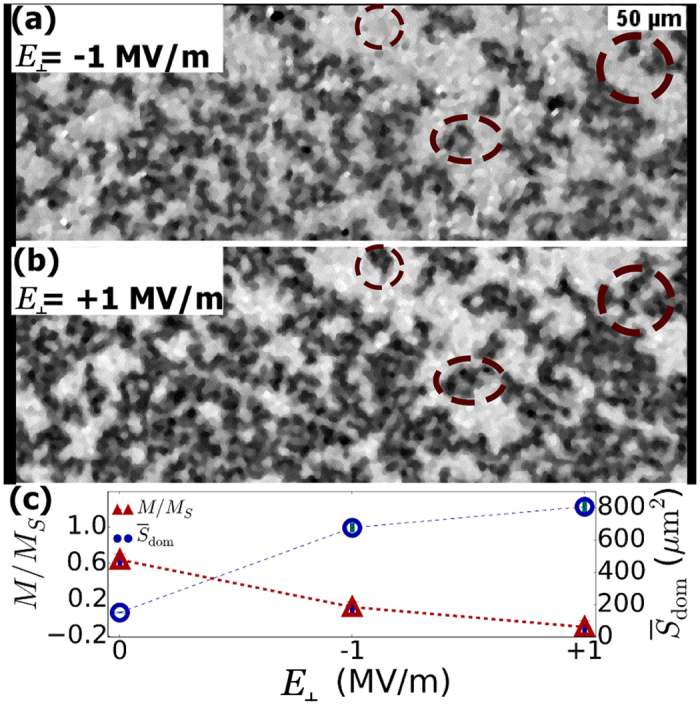
Kerr microscopy images of domain expansion under a modest out-of-plane magnetic field (−2.4 mT) and subsequent applications of electric fields to the PZT substrate of (**a**) −1 MV/m and (**b**) +1 MV/m (Circles in (**a,b**) highlight newly reversed areas due to electric field polarity switch). Changes in the magnetization (red triangles) and the average reversed (dark contrast) domain size (open blue circles) are shown in (**c**) under the series of applied electric fields.

Voltage-induced strains in the PZT can be used to propagate magnetic domain walls and reverse the magnetization. Figure 4 shows two Kerr microscopy images taken under a modest applied out-of-plane magnetic field (−2.4 mT) below *μ*_0_*H*_*c*_(−4.2 mT) after applying a large, saturating positive magnetic field (28.6 mT) and alternating the direction of a 1 MV/m out-of-plane electric field in the PZT. Prior to applying an electric field, some reversed domains have already nucleated and the reduced magnetization is stable at a value of *M*/*M*_*s*_ = 0.64 ± 0.06 (see Supplementary Information). However, following the application of a −1 MV/m electric field, we observe a significant increase in the area occupied by reversed magnetic domains seen in Fig. 4(a). This corresponds to a reduced magnetization of 0.12 +/ 0.06. Then, after reversing the polarity of the electric field to +1 MV/m, we show another moderate increase in reversed domains (Fig. 4(b)), which assists in reversing the net magnetization direction (*M*/*M*_*s*_ = −0.10 ± 0.07).

We also note that the size of reversed domains increases under applied electric fields. As shown in Fig. 4(c), the average reversed domain size, as determined by lineal analysis of the pictures in Fig. 4(a,b), increases from 152 ± 4 μm^2^ before applying an out-of-plane electric field, to 680 ± 30 μm^2^ after applying −1 MV/m. This increases again to 810 ± 40 μm^2^ after reversing the polarity to +1 MV/m^28^. This demonstrates that domain expansion, as a consequence of domain wall de-pinning, can be enhanced by strain. This is in good agreement with previous studies that have demonstrated that strains in PMA films can enhance domain wall propagation by reducing the perpendicular anisotropy^9^,^11^.

### Lowering the reversal barrier with strain modification

Finally, we demonstrate proof-of-concept for this system for electrically-assisted switching of the magnetization. In Fig. 3(a) we saw that the magnetic coercivity of the Co/Ni multilayers depends on the electric field applied to the PZT. The coercivity can be reduced to *μ*_0_*H*_*c*_ = 3.5 ± 0.1 mT for *E*_⊥_ = −2 MV/m compared with *μ*_0_*H*_*c*_ = 4.6 ± 0.1 mT for *E*_⊥_ = +1 MV/m, a coercivity reduction of more than 30%. The electric-field modulation of the coercivity in the Co/Ni multilayers could be used to switch the magnetization.

**Figure 5:**
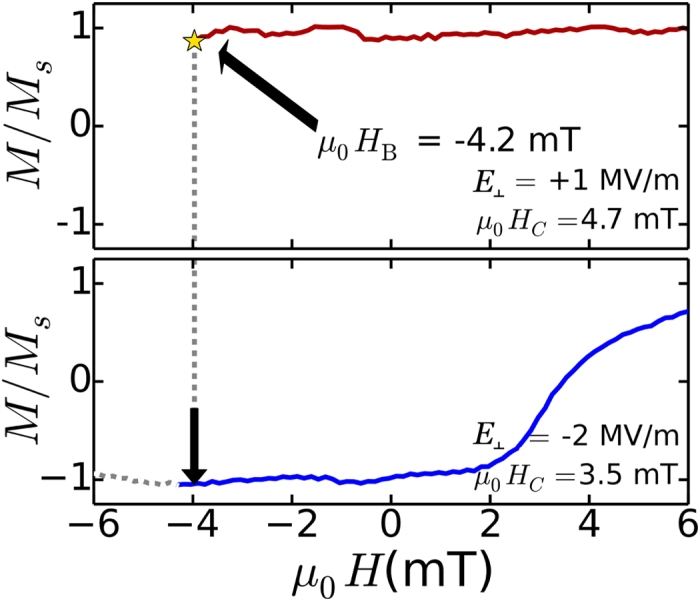
Concept illustrating voltage-assisted magnetization reversal. An out-of-plane bias magnetic field (−4.2 mT) is applied to a Co/Ni multilayered film on a PZT substrate under +1 MV/m electric field bias after saturation in a large positive magnetic field (red curve). Changing the electric bias to −2 MV/m drives the Co/Ni film into the low coercivity operating region, indicated by the arrow extending down from the yellow star. The Co/Ni film would subsequently follow the hysteresis curve defined by the low coercivity state (blue line).

Figure 5 illustrates a concept in which strain-assisted magnetization reversal might be implemented. An out-of-plane bias magnetic field (*μ*_0_*H*_*b*_ = −4.2 mT) is applied to a Co/Ni multilayered film on a PZT substrate under +1 MV/m out-of-plane electric field bias after saturation in a large positive magnetic field. This can be seen as the red solid line extending from large positive field down to −4.2 mT and denoted by a yellow star. Changing the electric bias to −2 MV/m drives the Co/Ni film into the low coercivity operating region, indicated by the arrow extending down from the yellow star. The Co/Ni film would subsequently follow the hysteresis curve defined by the low coercivity state, represented by the blue curve. For device implementation, the strain would be modified locally with voltages applied to a contact region of the piezoelectric element directly adjacent or underneath the magnetic film or nanostructure. Furthermore, it would be desirable to transfer larger in-plane strains, which could be implemented by in-plane poling of a thin-film ferroelectric underlayer such as PMN-PT grown under the magnetic film^9^. In such a geometry, a modest increase of the in-plane strain to 0.3% would reduce the PMA energy of a 3 nm thick, 30 nm diameter nanomagnet by 5 k_B_T (Δ*E*_*elastic*_ × *V*), which would significantly reduce the height of the energy barrier for magnetization reversal. While scaling these materials to device-relevant dimensions (i.e. nanoscale) may raise new issues^29^,^30^, the demonstrated ability to use electric fields to modify the reversal of an ultrathin film with broadly tunable PMA is a necessary step towards the design of these new magnetic devices. This could enable a non-volatile strain-based memory, in which the non-volatility of high PMA magnets is combined with the low power consumption of magnetoelectric control. It may also be possible to utilize this system for strain-assisted magnetic recording applications, in which reversible electric field manipulation of the magnetic coercivity could provide an alternative to lower power information storage^31^,^32^.

## Discussion

In summary, we have exploited the tunable perpendicular magnetic anisotropy in Co/Ni multilayered films and elastic coupling to a PZT substrate for the development of voltage-assisted magnetization reversal. This hybrid ferroelectric-ferromagnetic system exhibits strong PMA, sufficient for producing sub-30 nm diameter, ultrathin magnetic nano-objects with good thermal stability. The magnetoelectric coupling in this system that can reduce the Co/Ni coercive field is apparently strain mediated through lateral compression generated in the PZT, evidenced by the nearly identically non-linear strain-voltage and magnetic coercivity-voltage trends. We estimate that expansion of the PZT by 0.15% mediates a small reduction in the Co/Ni PMA energy due to increased magnetoelastic cost of the perpendicular magnetization under out-of-plane tensile strain in the Co/Ni multilayers. This PMA reduction was followed by a pronounced change in the Co/Ni coercive field of greater than 30%, which could potentially be used for voltage-assisted magnetization reversal applications. Finally, we proposed a concept for the implementation of a strain-mediated magnetization reversal, in which an applied voltage is used to lower a Co/Ni nanomagnet’s coercivity below a moderate applied bias magnetic field. Therefore, high PMA Co/Ni multilayers combined with a PZT substrate are a promising materials system for voltage-controlled magnetic data storage and spintronics applications, in which the non-volatility of the PMA material is combined with the voltage-enabled reduction in switching energy.

## Methods

One-mm-thick Pb(Zr_0.52_Ti_0.48_)O_3_ (PZT) plates were obtained from DeL Piezo Specialties, LLC (West Palm Beach, Florida, USA). Typical surface roughness of at least 100 nm *rms* was reduced to 3 nm *rms* by using a chemical-mechanical polishing (CMP) tool (Bruker CP-4) at the NIST Nanofabrication Facility. The PZT plates were polished using the following CMP recipe module: 5 minutes under 4 psi (28 kPa) with a 60 mL/minute flow of a 1:5 silica slurry (Eminess 556 Colloidal Silica) to distilled water dilution followed by a 2 minute rinse under 1 psi (7 kPa) with a 90 mL/minute flow of distilled water. This cycle was repeated eight times, after which the PZT plate was promptly transferred from the CMP wafer holder to bath of a dilute ammonia solution heated to 80 degrees C for five minutes, followed by a distilled water soak with ultrasonic agitation for three minutes. This post-CMP process was critical to the removal of remaining slurry particles that would otherwise adhere to the PZT surface.

Plates were processed into smaller pieces using a semi-automated dicing saw. After spin-coating a thin layer of Shipley 1813 (MicroChem Corp., Westborough, Massachusetts, USA) photoresist onto the surface to protect the surface during dicing, 5 mm × 5 mm substrate pieces were diced from a polished PZT plate. The pieces were subsequently cleaned using ultrasonic agitation in acetone for 5 minutes, followed by 5 minutes of ultrasonic agitation in isopropanol, and a rinse in distilled water, followed by drying with compressed air.

Magnetic multilayered films were grown on the substrate pieces using dc magnetron sputtering. Following a 5 minute clean under low energy Ar^+^ ion bombardment (500 eV) to remove organic contamination, Co/Ni multilayered films of varying thicknesses were grown with identical seed layers, starting with an amorphous 3 nm Ta adhesion layer followed by 2 nm Pt to encourage (111) face-centered-cubic growth of the subsequent film (see Supplementary Material for structural characterization). Multilayered films all had the same capping sequence, with 3 nm Ta on top of 1.6 nm Pt. Five different Co/Ni multilayers were synthesized: [Co(0.15)/Ni(0.3)]_x16_; [Co(0.2)/Ni(0.4)]_x16_; [Co(0.2)/Ni(0.6)]_x16_; [Co(0.15)/Ni(0.6)]_x16_; [Co(0.15)/Ni(0.6)]_x4_ where all thicknesses are given in nanometers. For symmetry purposes, each Co/Ni multilayered film is additionally topped with an extra cobalt layer of the same thickness as in the multilayer (e.g [Co(0.15)/Ni(0.6)]_x4_ has an added 0.15 nm cobalt layer before the Pt/Ta capping bilayer). Deposition rates and thicknesses were recorded using two parallel calibrated quartz crystal monitors. Film thicknesses varied by approximately five percent.

Magnetic measurements were conducted using a vibrating sample magnetometer (VSM, MicroSense, Lowell, MA, USA), ferromagnetic resonance (FMR) spectrometer, and a magneto-optic Kerr effect (MOKE) microscope. Magnetization versus applied magnetic field measurements were measured using VSM to determine saturation magnetization and to identify easy and hard axes of magnetization. Acquisition of a background for subtraction of the diamagnetic substrate signal was accomplished from measurement of an identical 5 mm × 5 mm substrate cleave without the magnetic multilayered film. FMR spectroscopy was conducted on a grounded coplanar waveguide (GCPWG), implementing a 40 GHz zero-bias diode detector (Krytar, Sunnyvale, CA, USA) and a 50 GHz *rf* current source (Agilent HP86530, Keysight, Santa Rosa CA, USA). Using a fixed *rf* current amplitude (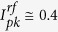 mA) and frequencies between 10 GHz and 30 GHz, the magnetic field from a dc electromagnet was swept between 24 kA/m and 1000 kA/m. A pair of secondary coils embedded in the dc electromagnet provides a low frequency alternating field (*f* = 177 Hz, 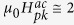 mT) for lock-in detection of the differential power absorbed by the GCPWG and sample as the dc magnetic field is swept. The applied dc and ac fields were monitored with a Hall probe sensor. MOKE microscopy was conducted with a 10x magnifier and two polarizing lenses whose polarizations are 2–5 degrees away from orthogonal. Applied dc fields in the MOKE microscope were generated with an electromagnet, and were correlated back to currents in the electromagnet using a Hall probe sensor (Lake Shore Cryotronics, Westerville, OH, USA).

Certain equipment, instruments or materials are identified in this paper in order to adequately specify the experimental details. Such identification does not imply recommendation by the National Institute of Standards and Technology nor does it imply the materials are necessarily the best available for the purpose.

## Additional Information

**How to cite this article**: Gopman, D. B. *et al*. Strain-assisted magnetization reversal in Co/Ni multilayers with perpendicular magnetic anisotropy. *Sci. Rep.* **6**, 27774; doi: 10.1038/srep27774 (2016).

## Supplementary Material

Supplementary Information
