# Supplementary material for: Strain-assisted magnetization reversal in Co/Ni multilayers with perpendicular magnetic anisotropy
Source: Sci Rep. 2016 Jun 14;6:27774. doi: 10.1038/srep27774 (PMC4906286; doi:10.1038/srep27774)
Supplement: Supplementary Information [file srep27774-s1.pdf]

Supplementary Information

Strain-assisted magnetization reversal in Co/Ni multilayers with perpendicular magnetic anisotropy

Gopman, D. B.[1,*], Dennis, C. L.[1], Chen, P. J.[1], Iunin, Y. L.[1, 2], P. Finkel[3], M. Staruch[3], and Shull, R. D.[1]

[1]Materials Science and Engineering Division, NIST, Gaithersburg, MD, 20899, USA
[2]Institute of Solid State Physics, RAS, Chernogolovka, 142432, Russia
[3]U.S. Naval Research Laboratory, Washington, DC, 20375, USA

I. Structural properties of Co/Ni multilayers grown on PZT

Following chemical-mechanical planarization, the PZT substrate surface roughness had been reduced from hundreds of nanometers to 3.6 ± 0.2 nm *rms*. At this degree of planarization, the underlying grain structure of this polycrystalline, piezoelectric ceramic was evident, with grain sizes ranging from hundreds of nm to several microns, as shown by the atomic force micrograph of the PZT substrate prior to deposition in Fig. S1.

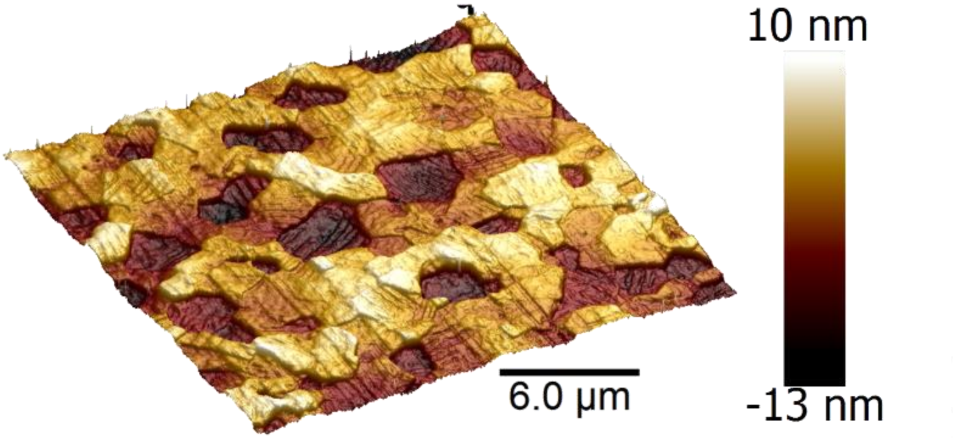

Figure S1: Atomic force micrograph of the PZT substrate after chemical-mechanical planarization but prior to film deposition.

After planarization of the PZT substrate, the following magnetic multilayered film was grown using sputter deposition (thicknesses in parentheses are indicated in nm): Ta(3)/Pt(2)/[Co(0.15)/Ni(0.6)]$_{x4}$/Co(0.15)/Pt(1.6)/Ta(3). The roughness of the top surface of the magnetic film was also investigated using atomic force microscopy, as shown in Fig. S2. The calculated roughness of this surface was 1.5 ± 0.2 nm *rms*. Compared with the relatively higher roughness of the PZT substrate (3.6 ± 0.2 nm *rms*), this value indicates a planarizing effect as the sputtered film fills the voids between PZT grains. Also noted in the main text, this moderate interfacial roughness limits the coherent strain transfer between PZT and the multilayered film, which may be mitigating the strength of magnetoelectric effects in this system.

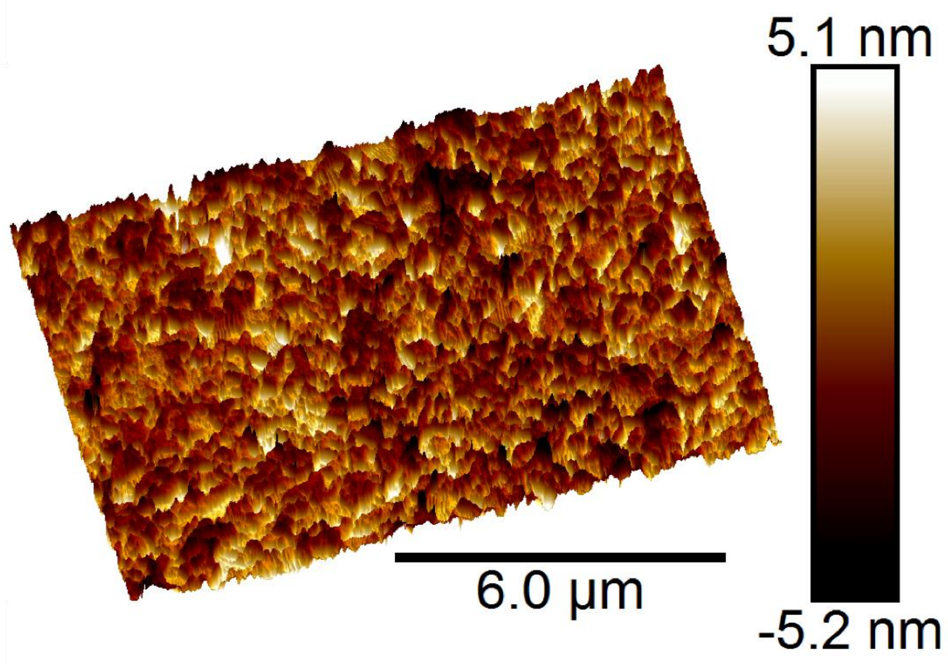

Figure S2: Atomic force micrograph of the sputtered Co/Ni multilayered film on top of the PZT substrate.

Consequently, the random orientation of the PZT grains permitted the full range of allowed reflections under x-ray diffraction. Figure S2 shows a range of reflections for the PZT substrate prior to coating with the magnetic multilayered film. The peaks are labeled with their corresponding reflection plane.

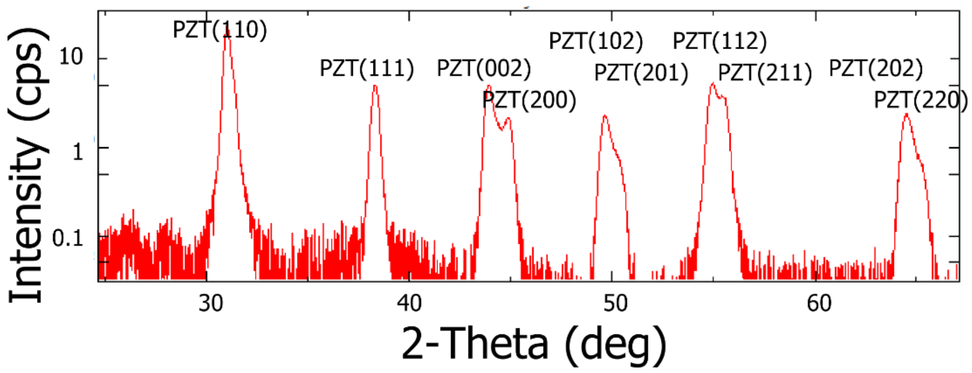

Figure S3: X-ray diffraction of PZT substrate prior to film deposition. Peak positions are marked with crystallographic orientation and material.

X-ray diffraction was again recorded on the film + substrate complex. In Fig. S4, we show the Intensity (in counts per second, cps) versus 2-Theta angle. We immediately notice that all of the peaks in Fig. S4 are present in this scan, with the addition of three added peaks, corresponding to the face-centered cubic peaks: Pt(111), Co/Pt(111) and Co/Ni(111). We see no evidence of Pt(220) or Pt(200) peaks, which suggests that most of the grains are oriented (111) out-of-the-plane, which also agrees with the magnetic measurements showing strong perpendicular magnetic anisotropy (correlated to fcc (111) texture in Co/Ni).

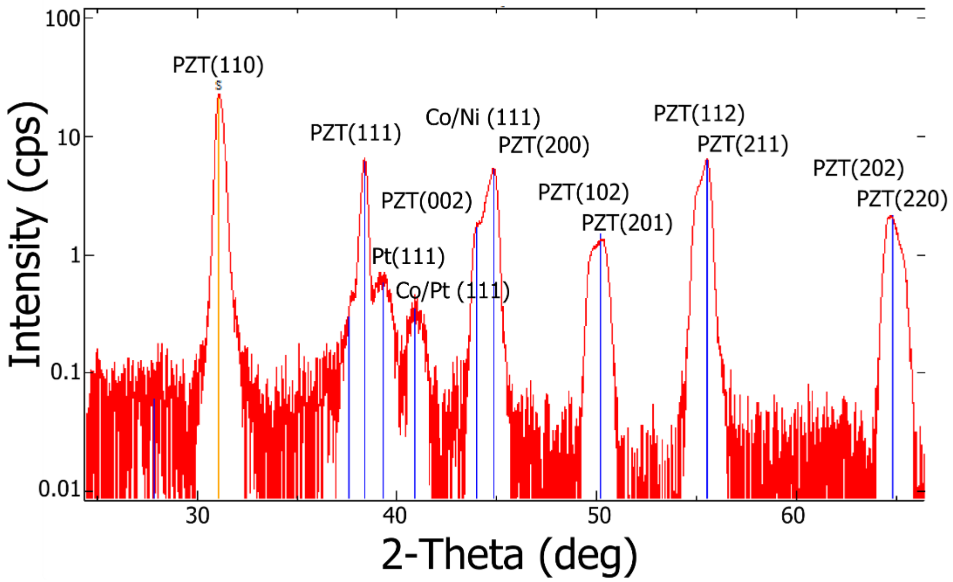

Figure S4: X-ray diffraction of Co/Ni multilayers on PZT. Peak positions are marked with crystallographic orientation and material.

II. MOKE Microscopy of Domain Propagation

The magnetization and average domain size are calculated by first obtaining a magnetic background and thresholding the image as regions of binary up and down domains. Figure S5 shows a topographic image of the surface of the film under saturating field conditions (30 mT out-of-plane applied magnetic field). This figure presents a fixed topographic background that we can subtract to enhance the magnetic Kerr contrast of the Co/Ni film.

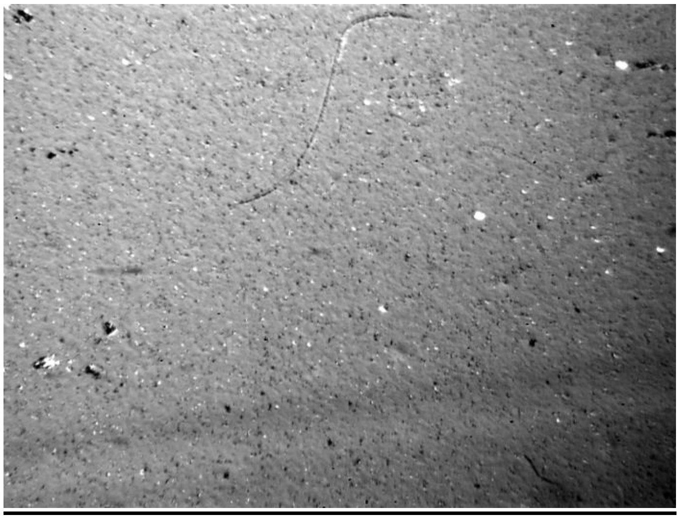

Figure S5: Topographical Image of Co/Ni Multilayered film under saturating magnetic field.

In Figs. S6-S8, we can see that the magnetic contrast is enhanced after subtracting the non-magnetic (topographic) background from images taken under non-saturating conditions. We

note that the presence of topographic defects, such as scratches in the film surface, may lead to a local enhancement of domain reversal in the region surrounding this topography. In particular, the scratch seen in the top-center of topographic image Fig. S4 tends to exhibit a larger density of reversed domains in Figs. S6-S8 under applied magnetic fields and applied electric fields.

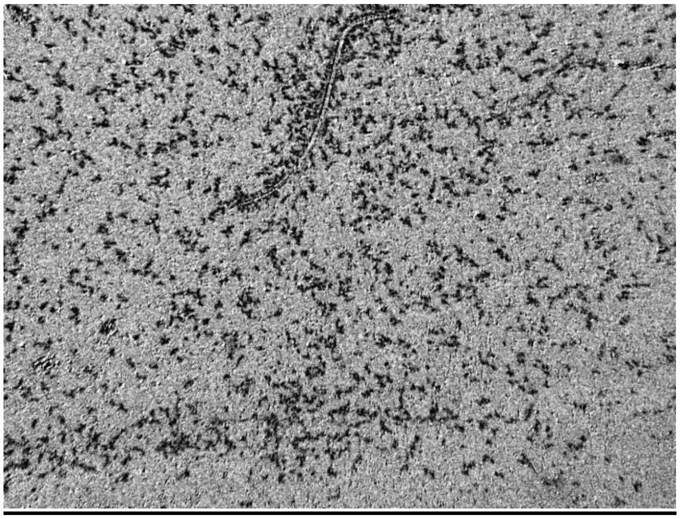

Figure S6: Magnetic contrast image of Co/Ni Multilayered film under small, negative out-of-plane field (-2.4 mT) and zero applied electric field.

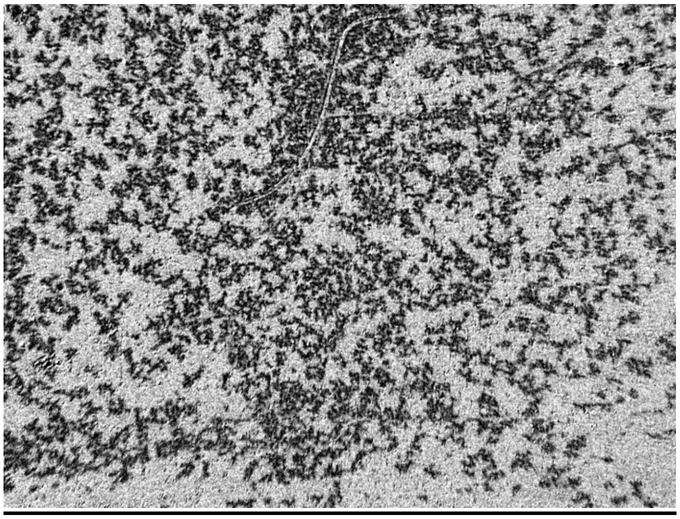

Figure S7: Magnetic contrast image of Co/Ni Multilayered film under small, negative out-of-plane field (-2.4 mT) and -1 MV/m electric field applied between the Co/Ni multilayered film and the back electrode of the PZT substrate.

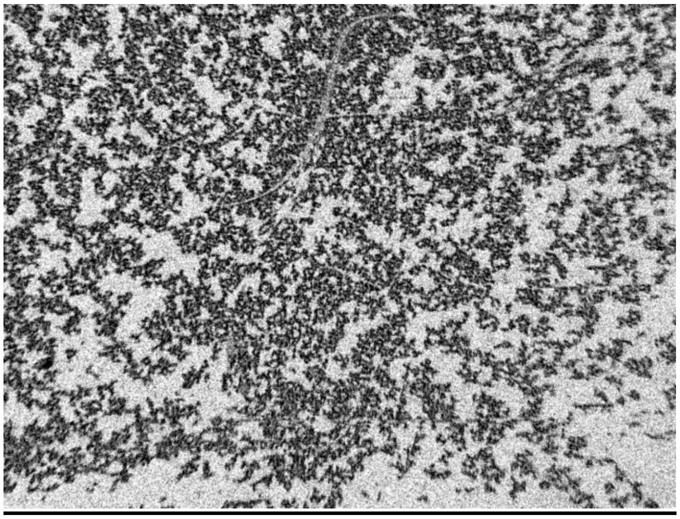

Figure S8: Magnetic contrast image of Co/Ni Multilayered film under small, negative out-of-plane field (-2.4 mT) and +1 MV/m electric field applied between the Co/Ni multilayered film and the back electrode of the PZT substrate.
